# Data-based modeling analysis of transfusion-transmitted malaria

**DOI:** 10.1016/j.idm.2026.09.005

**Published:** 2026-09-26

**Authors:** Kaihui Liu, Wen Shi

**Affiliations:** School of Mathematical Science, Jiangsu University, Zhenjiang, Jiangsu Province, China

**Keywords:** Transfusion-transmitted malaria, Mosquito-borne transmission, Basic reproduction number, MCMC parameter estimation, Nigeria

## Abstract

Malaria is one of leading causes of morbidity and mortality in sub-Saharan Africa, with Nigeria bearing the highest global burden. Although vector-borne transmission through female *Anopheles* mosquitoes is well-characterized, increasing evidence of malaria parasite prevalence among asymptomatic blood donors uncover that transfusion-transmitted malaria cannot be ignored. This paper proposes a compartmental model that integrates both conventional mosquito-borne transmission and transfusion-transmitted pathways. Based on this model, contributions of transfusion-mediated transmission to the basic reproduction number (*R*_0_) and new infection flows are quantified rigorously. Key parameters related to malaria transmission are estimated via Markov Chain Monte Carlo (MCMC) methods utilizing malaria incidence data of Nigeria from 2015 to 2024 years. The model accurately fits the observed data. Sensitivity analysis using Partial Rank Correlation Coefficients shows that *R*_0_ is highly sensitive to the transfusion-transmitted rate (*σ*_*t*_), highlighting the epidemiological importance of blood safety. Time-series and bivariate contour analyses further demonstrate that both mosquito-borne and transfusion-mediated transmission drastically elevate the outbreak potential. Route-specific and counterfactual contribution analyses show that mosquito-borne transmission remains the main driver of malaria persistence while transfusion-mediated transmission can substantially increase cumulative infections. Notably, eliminating transfusion-mediated transmission may prevent more cases than its direct contribution suggests, highlighting the indirect epidemiological impact of transfusion-mediated pathway. These findings support integrated malaria control strategies combining sustained vector control with strengthened blood-safety measures, especially in endemic regions with high transfusion demand and limited donor screening.

## 1 Introduction

Malaria remains one of the most significant threats to global public health, particularly in tropical and subtropical regions. According to the report of World Health Organization (WHO), an estimated 282 million malaria cases were reported across 80 endemic countries in 2024, representing an increase of 9 million cases compared to 2023 (WHO, 2025a). Nigeria contributes disproportionately to this burden, accounting for approximately 30.3% of global malaria cases and 38.6% of malaria deaths (WHO, 2025a). As documented by the WHO Malaria Dashboard, malaria incidence in Nigeria saw a decrease by approximately 25.6% from 2000 to 2015 years (WHO, 2025b). This declining trend was followed by an approximately 8.5% increase during next decade, i.e., between 2015 and 2024 years (WHO, 2025b). This unexpected resurgence, despite sustained vector-control interventions, suggests that additional transmission pathways may be undermining elimination efforts.

Traditional researches of malaria transmission emphasize the vector-borne route, wherein infected female *Anopheles* mosquitoes transmit *Plasmodium* parasites to susceptible human hosts during blood meals (Sollelis et al., 2024). This biological pathway has been extensively characterized through mathematical modeling, leading to important insights regarding intervention strategies and transmission dynamics (Adegbite et al., 2023; Rajnarayanan et al., 2025; Grosso et al., 2025; Lou & Zhao, 2010, 2011). However, another transmission pathway, i.e., transfusion-transmitted malaria has received comparatively little attention in both epidemiological modeling and public health policy, particularly in endemic regions (Loua et al., 2024; Lima et al., 2018). Transfusion-transmitted malaria occurs when whole blood or blood components containing *Plasmodium*-infected erythrocytes are transfused from an asymptomatic donor to a susceptible recipient (Ahmadpour et al., 2019). Unlike mosquito-borne transmission, transfusion-transmitted malaria bypasses the hepatic stage of the parasite life cycle, directly introducing blood-stage parasites into the circulation of the recipient (Malede et al., 2026). This can result in more severe clinical outcomes, especially in vulnerable populations including neonates, pregnant women, surgical patients, and immuno-compromised individuals (Antwi-Baffour et al., 2019).

The magnitude of transfusion-transmitted risk is substantial in malaria-endemic sub-Saharan Africa (Loua et al., 2024). Cross-sectional studies have documented high malaria parasite prevalence among asymptomatic blood donors across multiple Nigerian settings. A study in Enugu metropolis reported a malaria prevalence of 39.3% among 377 blood donors, with the highest rates observed in the 16 − 35 year age groups (Aluh et al., 2025). Similarly, research in southeastern Nigeria found a prevalence of 40.9% among 325 donors, with *Plasmodium falciparum* identified in all positive cases (Uneke et al., 2006). A systematic review and meta-analysis found that nearly one-third of Nigerian blood donors tested positive for malaria parasites (Ahmadpour et al., 2019). This conclusion is reinforced by other individual studies. One cross-sectional investigation at a tertiary hospital reported a malaria prevalence of 21.1% among donors, with commercial donors having the highest rate (33.3%) (Olopade et al., 2024).

Against the high prevalence of asymptomatic parasitemia among blood donors, many institutions have yet to incorporate malaria parasite screening into their pre-donation protocols (Dauda & Mohammed, 2017). According to Nigeria's operational guidelines, malaria parasitemia is not included in the mandatory screening tests of donated blood, which only cover human immunodeficiency virus (HIV), hepatitis B virus (HBV), hepatitis C virus (HCV) and syphilis (National Blood Service Commission., 2021). Consequently, screening practices for malaria parasites may vary across blood-transfusion facilities. In view of high prevalence of asymptomatic parasitemia among blood donors, the gap between policy and implementation may generate a persistent residual risk of transfusion. Therefore, quantifying its direct and indirect epidemiological contributions may provide additional insights into malaria persistence and help explain why conventional mosquito-focused measures alone have failed to sustain the earlier decline.

Despite this evident risk, mathematical models of malaria transmission have predominantly focused on vector-borne pathways. Recent efforts have begun to address this gap. Akowe et al. developed a novel model integrating vector and non-vector transmission routes, including blood transfusions, congenital transmission, and healthcare-associated transmission (Akowe et al., 2025). Their results indicated that non-vector pathways pose substantial risks even in regions with effective mosquito control (Akowe et al., 2025). Adeniyi et al. specifically examined transfusion-transmitted malaria using a SIR framework, deriving a transfusion-specific reproduction number and demonstrating that blood transfusion parameters significantly influence overall transmission dynamics (Adeniyi et al., 2023). However, existing models exhibit several limitations. First, previous models treat transfusion transmission as a simple additive term without explicitly modeling the role of asymptomatic carriage. Second, parameter estimation for transfusion-related parameters has been limited in relying on assumed values rather than data-driven calibration. Third, the relative contribution of transfusion versus vector pathways to overall transmission and the implications the prioritization of control strategy remain inadequately quantified.

This paper addresses these gaps by proposing a compartmental model that explicitly incorporates both mosquito-borne and transfusion-mediated pathways. The model captures distinct mechanisms of each transmission route, including the role of asymptomatic infected individuals as blood donors. Rigorous analyses of the contributions of each transmission pathway are established based on this model. Using clinical malaria cases from Nigeria, we employ an adaptive Markov Chain Monte Carlo (MCMC) method to estimate key transmission parameter values. Numerical simulations further compare model outputs with observed data, assess parameter sensitivity, and evaluate the relative contributions of transfusion-mediated transmission to the basic reproduction number and new infection flows.

## 2 Materials and methods

### 2.1 Mathematical model

The classical SEIR vector-host compartmental model is extended to capture the dual transmission routes of malaria, which are vector-borne transmission and transfusion-transmitted pathways. The model explicitly distinguishes two distinct exposed states in humans according to the route of infection: vector-borne exposure and transfusion-related exposure. Moreover, it differentiates asymptomatic carriers (who can transmit the parasite through blood donation) from symptomatic infectious individuals. The model formulation aims to quantify the contribution of asymptomatic blood donors to overall malaria transmission and to evaluate intervention strategies targeting both pathways.

The human population is stratified into susceptible humans (*S*_*h*_), exposed humans infected through mosquito-borne transmission (*E*_*hm*_) or via transfusion of infected blood (*E*_*ht*_), asymptomatic humans (*A*_*h*_) who serve as the primary source of transfusion-transmitted malaria when they donate blood, infected humans (*I*_*h*_) and recovered humans (*R*_*h*_). The total human population is denoted by *N*_*h*_ and defined as

$$N_{h}=S_{h}+E_{hm}+E_{ht}+A_{h}+I_{h}+R_{h}.$$

The mosquito population is divided into susceptible (*S*_*m*_), exposed (*E*_*m*_) and infectious (*I*_*m*_) mosquitoes. Let *N*_*m*_ denote the total mosquito population, which equals

$$N_{m}=S_{m}+E_{m}+I_{m}.$$

Susceptible humans can be infected with malaria through two different routes. One route is the classic mosquito-borne transmission, that is, susceptible humans can become exposed (*E*_*hm*_) by the bites of infectious mosquitoes. The force of infection for mosquito-borne transmission is

$$\lambda_{hm}=\sigma_{m}\frac{I_{m}}{N_{h}},$$

where *σ*_*m*_ is the transmission probability of malaria parasite from infectious mosquitoes to susceptible humans. Alternatively, susceptible humans can acquire the malaria parasite through transfusion of infected blood from asymptomatic humans and transfer to exposed (*E*_*ht*_) states.

The incidence of transfusion-transmitted exposure is modeled based on the following two assumptions. First, within a local stable blood-supply system, the rate at which potentially contaminated blood units enter the blood-supply system is assumed to be proportional to the number of asymptomatic parasitemic individuals who participate in blood donation. Let *ρ* denote the effective fraction of asymptomatic parasitemic individuals contributing potentially infectious blood donations. Then, *ρA*_*h*_(*t*) represents the effective asymptomatic infectious donor pool. Second, the demand for transfusion is approximately proportional to the size of susceptible recipients, *S*_*h*_(*t*).

The rate of effective infectious donor-recipient matching can be approximated by the bilinear term *ρA*_*h*_(*t*)*S*_*h*_(*t*) when donor-side supply and recipient-side demand are combined through the local blood collection, storage, and allocation system. Then, the force of infection for transfusion-mediated transmission is modeled as

$$\lambda_{ht}=\sigma_{t}\rho A_{h},$$

where *σ*_*t*_ with units person^−1^time^−1^, is a composite effective transfusion-mediated transmission coefficient per asymptomatic donor-susceptible recipient pair, incorporating the rate at which susceptible recipients receive blood, donor-recipient matching within the blood-supply system, the probability that an infected blood unit escapes screening or pathogen-reduction procedures, and the probability that transfusion of an infected unit establishes malaria infection.

After the incubation period, exposed humans progress to either asymptomatic (*A*_*h*_) or symptomatic (*I*_*h*_) state, with probabilities *p* and 1 − *p*, respectively. Both asymptomatic and symptomatic individuals can transmit malaria parasite through the bites of susceptible mosquitoes. The force of infection for mosquitoes is

$$\lambda_{m}=\kappa \frac{I_{h}+A_{h}}{N_{h}},$$

where *κ* is the effective contact rate of susceptible mosquitoes with infected humans through biting. In addition to the natural death, symptomatic infectious humans can die from severe malaria. Then, these infected humans recover from malaria and transfer into recovered state (*R*_*h*_). Due to the complexity of malaria, these recovered individuals can become susceptible and reinfect when the acquired parasite strain-specific immunity is waning at a rate of *ξ*. The transmission diagram of malaria parasite between mosquitoes and humans is shown in Fig. 1.

**Fig. 1:**
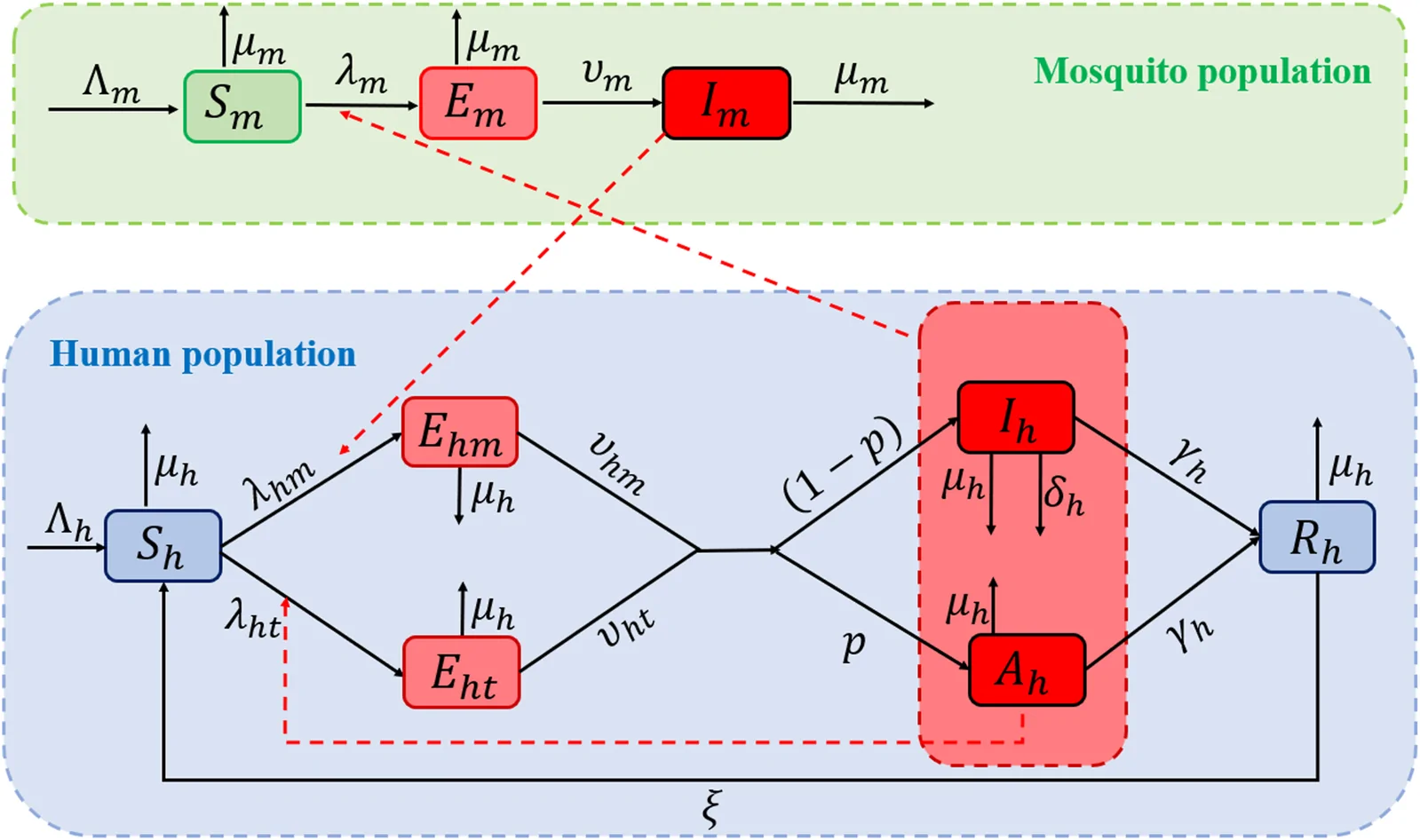
Schematic flow chart of the malaria transmission.

Based on the above assumptions and model diagram in Fig. 1, the following system of equations is established to describe the transmission dynamics of malaria parasite between mosquitoes and humans, incorporating both mosquito-borne and transfusion-mediated transmission. Detailed descriptions of parameters in model (2.1) are illustrated in Table 1.

(2.1) $$\left\{\begin{array}{ll} \frac{dS_{m}}{dt} & =\Lambda_{m}-\kappa \frac{I_{h}+A_{h}}{N_{h}}S_{m}-\mu_{m}S_{m},\\ \frac{dE_{m}}{dt} & =\kappa \frac{I_{h}+A_{h}}{N_{h}}S_{m}-\nu_{m}E_{m}-\mu_{m}E_{m},\\ \frac{dI_{m}}{dt} & =\nu_{m}E_{m}-\mu_{m}I_{m},\\ \frac{dS_{h}}{dt} & =\Lambda_{h}-\sigma_{m}\frac{I_{m}}{N_{h}}S_{h}-\sigma_{t}\rho A_{h}S_{h}-\mu_{h}S_{h}+\xi R_{h},\\ \frac{dE_{hm}}{dt} & =\sigma_{m}\frac{I_{m}}{N_{h}}S_{h}-\nu_{hm}E_{hm}-\mu_{h}E_{hm},\\ \frac{dE_{ht}}{dt} & =\sigma_{t}\rho A_{h}S_{h}-\nu_{ht}E_{ht}-\mu_{h}E_{ht},\\ \frac{dA_{h}}{dt} & =p\nu_{hm}E_{hm}+p\nu_{ht}E_{ht}-\left(\mu_{h}+\gamma_{h}\right)A_{h},\\ \frac{dI_{h}}{dt} & =\left(1-p\right)\nu_{hm}E_{hm}+\left(1-p\right)\nu_{ht}E_{ht}-\left(\mu_{h}+\delta_{h}+\gamma_{h}\right)I_{h},\\ \frac{dR_{h}}{dt} & =\gamma_{h}\left(I_{h}+A_{h}\right)-\mu_{h}R_{h}-\xi R_{h}. \end{array}\right)$$

**Table 1:** Biological explanations for parameters of model (2.1).

Para.	Description
Λ_*h*_	Recruitment rate of humans
*μ*_*h*_	Per-capita natural death rate of humans
*γ*_*h*_	Recovery rate of infectious humans
*ν*_*hm*_	Disease progression rate of exposed humans infected by mosquito bites
*ν*_*ht*_	Disease progression rate of exposed humans infected through blood transfusion
Λ_*m*_	Recruitment rate of mosquitoes
*μ*_*m*_	Per-capita natural death rate of mosquitoes
*ν*_*m*_	Progression rate of mosquitoes from exposed to infectious state *δ*_*h*_ Malaria induced death rate
*ρ*	Effective fraction of asymptomatic individuals contributing potentially infectious blood donations
*p*	Proportion of asymptomatic infections
*κ*	Effective contact rate of susceptible mosquitoes with infected humans through mosquito bites.
*σ*_*m*_	Effective contact rate of susceptible humans with infected mosquitoes
*σ*_*t*_	Effective transfusion-mediated transmission coefficient per susceptible recipient-asymptomatic donor pair, incorporating probabilities of transfusion demand, donor-recipient matching, screening failure, and transmission following transfusion.
*ξ*	Human immune waning rate

### 2.2 Contribution analysis of transfusion-mediated transmission

The inclusion of blood transfusion as an additional route of malaria transmission in this model may reshape the understanding of disease persistence and control, especially in areas where transfusion demand is high and blood-screening systems are inadequate. Contribution analysis therefore provides a rigorous means to determine the extent of transfusion-mediated transmission contributing to malaria incidence compared with mosquito-borne transmission. To quantify the relative roles of the transfusion-mediated transmission routes, the total basic reproduction number and new human infection flow are decomposed according to their two transmission components.

#### 2.2.1 Relative contribution to the basic reproduction number

The basic reproduction number, denoted by *R*_0_, quantifies the expected number of secondary cases generated by a single infected individual in a fully susceptible population. It is well-known that *R*_0_ serves as the threshold to determine whether the disease invade and persist in the population. By applying the method of next generation matrix (van den Driessche & Watmough, 2002), we obtain the following explicit expression for *R*_0_,

(2.2) $$R_{0}=\frac{1}{2}\left(R_{0t}+\sqrt{R_{0t}^{2}+4R_{0m}^{2}}\right),$$

where

(2.3) $$R_{0m}=\sqrt{\frac{\kappa S_{m}^{*}}{N_{h}^{*}}\cdot \frac{\nu_{m}}{\mu_{m}+\nu_{m}}\cdot \frac{\sigma_{m}}{\mu_{m}}\cdot \left[p\frac{\nu_{hm}}{\mu_{h}+\nu_{hm}}\frac{1}{\mu_{h}+\gamma_{h}}+\left(1-p\right)\frac{\nu_{hm}}{\mu_{h}+\nu_{hm}}\frac{1}{\mu_{h}+\delta_{h}+\gamma_{h}}\right]},$$

can be defined as the reproduction number of mosquito-borne transmission, and

(2.4) $$R_{0t}=\sigma_{t}\rho S_{h}^{*}\cdot \frac{1}{\left(\mu_{h}+\gamma_{h}\right)}\cdot p\frac{\nu_{ht}}{\left(\mu_{h}+\nu_{ht}\right)},$$

can be defined as the reproduction number of transfusion-mediated transmission. Detailed derivation of the basic reproduction number is provided in the appendix.

These definitions are stems directly from the meaning of their components. Note that $\kappa S_{m}^{*}/N_{h}^{*}$ denotes the number of mosquitoes infected by one infectious human. *ν*_*m*_/(*μ*_*m*_ + *ν*_*m*_) is the probability that an exposed mosquito survives the latent period to become infectious. *σ*_*m*_/*μ*_*m*_ represents the expected number of human infections caused by one infected mosquito during its average lifespan 1/*μ*_*m*_. The term with square brackets in (2.3) describes an exposed human infected via mosquito bites (*E*_*hm*_) becoming either *A*_*h*_ or *I*_*h*_ and surviving from the respective period of infectiousness. Thus, *R*_0*m*_ measures the impact of mosquito-borne transmission pathway.

While *R*_0*t*_ indicates the influences of transfusion-mediated transmission, since $\sigma_{t}\rho S_{h}^{*}$ in (2.4) represents the average number of new human infections generated per unit time by one asymptomatic individual *A*_*h*_ via blood transfusion, 1/(*μ*_*h*_ + *γ*_*h*_) is the mean infectious period of an asymptomatic individual, and *pν*_*ht*_/(*μ*_*h*_ + *ν*_*ht*_) denotes the probability that an individual infected through blood transfusion will eventually become an asymptomatic carrier *A*_*h*_.

It can be derived from equation (2.2) that *R*_0_ satisfies the following quadratic relation:

$$R_{0}^{2}=R_{0t}R_{0}+R_{0m}^{2}.$$

Dividing both sides of the above equation by $R_{0}^{2}$, we obtain

$$1=\frac{R_{0t}}{R_{0}}+\frac{R_{0m}^{2}}{R_{0}^{2}}.$$

Therefore, the relative contributions of the mosquito-borne and transfusion-mediated transmission are defined as

(2.5) $$H_{t}=\frac{R_{0t}}{R_{0}}\;\;\;\;\;\text{and}\;\;\;\;\;H_{m}=\frac{R_{0m}^{2}}{R_{0}^{2}}$$

respectively. These two quantities satisfy

$$H_{m}+H_{t}=1,$$

and hence provide a normalized decomposition of the overall basic reproduction number.

#### 2.2.2 Relative contribution to new infection flows

From system (2.1), the force of infection acting on susceptible humans can be written as

$$\lambda_{h}\left(t\right)=\lambda_{m}\left(t\right)+\lambda_{t}\left(t\right),$$

where

$$\lambda_{m}\left(t\right)=\sigma_{m}\frac{I_{m}\left(t\right)}{N_{h}\left(t\right)},\;\lambda_{t}\left(t\right)=\sigma_{t}\rho A_{h}\left(t\right),$$

and

$$N_{h}\left(t\right)=S_{h}\left(t\right)+E_{hm}\left(t\right)+E_{ht}\left(t\right)+A_{h}\left(t\right)+I_{h}\left(t\right)+R_{h}\left(t\right).$$

Here, *λ*_*m*_(*t*) represents the mosquito-borne force of infection, while *λ*_*t*_(*t*) denotes the transfusion-mediated force of infection.

Since the actual inflow of newly infected humans depends on the number of susceptibles, we further define the route-specific new infection flows by

(2.6) $$J_{m}\left(t\right)=\lambda_{m}\left(t\right)S_{h}\left(t\right)=\sigma_{m}\frac{I_{m}\left(t\right)}{N_{h}\left(t\right)}S_{h}\left(t\right),$$

and

(2.7) $$J_{t}\left(t\right)=\lambda_{t}\left(t\right)S_{h}\left(t\right)=\sigma_{t}\rho A_{h}\left(t\right)S_{h}\left(t\right).$$

Thus, *J*_*m*_(*t*) and *J*_*t*_(*t*) measure the numbers of newly infected humans generated per unit time through mosquito bites and blood transfusion, respectively.

To assess the time-dependent relative importance of transfusion-mediated transmission, we introduce the instantaneous attributable fraction

(2.8) $$F_{t}^{\text{inst}}\left(t\right)=\frac{J_{t}\left(t\right)}{J_{m}\left(t\right)+J_{t}\left(t\right)}.$$

Similarly, the mosquito-borne attributable fraction is given by

(2.9) $$F_{m}^{\text{inst}}\left(t\right)=\frac{J_{m}\left(t\right)}{J_{m}\left(t\right)+J_{t}\left(t\right)},$$

with

$$F_{m}^{\text{inst}}\left(t\right)+F_{t}^{\text{inst}}\left(t\right)=1.$$

This quantity measures the proportion of all newly generated human infections at time *t* that is attributable to the transfusion pathway.

In addition, to evaluate the long-term burden associated with each transmission route, we compute the cumulative numbers of infections generated over the interval [0, *T*]:

(2.10) $$C_{m}\left(T\right)=\int_{0}^{T}J_{m}\left(t\right)\;dt,\;C_{t}\left(T\right)=\int_{0}^{T}J_{t}\left(t\right)\;dt.$$

Based on these quantities, we define the cumulative attributable fraction of transfusion-mediated transmission as

(2.11) $$F_{t}^{\text{cum}}\left(T\right)=\frac{C_{t}\left(T\right)}{C_{m}\left(T\right)+C_{t}\left(T\right)}.$$

Likewise, the cumulative attributable fraction of mosquito-borne transmission is

$$F_{m}^{\text{cum}}\left(T\right)=\frac{C_{m}\left(T\right)}{C_{m}\left(T\right)+C_{t}\left(T\right)}.$$

The quantity $F_{t}^{\text{cum}}\left(T\right)$ represents the proportion of the total cumulative infection burden over the study period that is attributable to blood transfusion.

To further quantify the epidemiological role of the transfusion route, we consider a counterfactual scenario in which transfusion-mediated transmission is removed by setting

$$\sigma_{t}=0.$$

Let *C*^full^(*T*) and *C*^no transfusion^(*T*) denote the cumulative total number of newly infected humans under the full model, and the counterfactual model without transfusion transmission respectively. Then the absolute number of infections attributable to the transfusion pathway is defined by

(2.12) $$\Delta C_{t}\left(T\right)=C^{\text{full}}\left(T\right)-C^{\text{no transfusion}}\left(T\right),$$

and the corresponding counterfactual attributable fraction is

(2.13) $$AF_{t}^{\text{cf}}\left(T\right)=\frac{C^{\text{full}}\left(T\right)-C^{\text{no transfusion}}\left(T\right)}{C^{\text{full}}\left(T\right)}.$$

This counterfactual quantity measures the proportion of total infection burden that would be averted if transfusion-mediated transmission were completely eliminated.

### 2.3 Data sources

Nigeria bears the heaviest malaria burden among nations globally. Beyond the dominant vector-borne route, Nigeria faces a secondary but non-negligible transmission pathway through unsafe blood transfusion. Several factors of Nigerian blood-transfusion system interact to cause the potential risk of transfusion-transmitted malaria. First, the demand for blood transfusion is enormous. It has been estimated that Nigeria requires a minimum of 1.8 million blood units annually to meet clinical needs (Iro et al., 2019). Second, the prevalence of asymptomatic *Plasmodium* parasitemia among blood donors is alarmingly high. Third, the fraction of blood units that escape malaria screening is substantial. Despite the WHO recommended that all donated blood should be tested for malaria where appropriate and possible, screening blood donations for malaria parasitemia is not mandatory in Nigerian health facilities. This regulatory omission, together with the high prevalence of asymptomatic parasitemia among donors may create a persistent transmission risk.

Nigeria malaria incidence data during the 2015-2024 period, extracted from the WHO Malaria Dashboard, is adopted for model calibration and parameter estimation. The raw dataset provides the mean number of new malaria incidence per 1, 000 population at risk per year. To apply this data to our model, we choose a hypothetical region with high malaria incidence in Nigeria. The average number of the population at risk in this region during these years is assumed as a stable total population of 200, 000 individuals, that is, $N_{h}^{*}=200,000$. Consequently, the total annual cases in this hypothetical region is obtained by scaling the original per 1, 000 population values by a factor of 200.

### 2.4 Parameter estimation method

Given the average life expectancy in Nigeria is approximately 55 years, the human mortality rate, *μ*_*h*_, is set as 1/(55 × 365), i.e., *μ*_*h*_ = 0.00005. Then, the human recruitment rate, Λ_*h*_ is calculated as $N_{h}^{*}\times \mu_{h}$, i.e., Λ_*h*_ = 10. The remaining model parameters including the human recovery rate (*γ*_*h*_), malaria induced death rate of infectious humans (*δ*_*h*_), mosquito recruitment (Λ_*m*_) and mortality rate (*μ*_*m*_) are fixed based on existing published literature, as detailed in Table 2. However, several critical parameters influencing malaria transmission dynamics could not be reliably determined from prior researches. In particular, the transmission rate via mosquito bites (*σ*_*m*_) or blood transfusion (*σ*_*t*_), effective contact rate of susceptible mosquitoes with infected humans (*κ*), the rate of immunity loss (*ξ*), the fraction of asymptomatic individuals among infected humans (*p*) and the proportion of asymptomatic individuals involving blood transfusion (*ρ*) are treated as unknown quantities to be estimated.

**Table 2:** Parameters values and their sources.

Para.	Value	Range	Reference
Λ_*h*_	10	[9.96, 10.32]	WHO (n.d.)
*μ*_*h*_	0.00005	[4.98, 5.16] × 10^−5^	WHO (n.d.)
*ν*_*hm*_	0.04	[0, 0.8]	Akowe et al. (2025), Adeniyi et al. (2023)
*ν*_*ht*_	0.04	[0, 0.8]	Akowe et al. (2025)
*γ*_*h*_	0.01	[0, 0.997]	Adeniyi et al. (2023), Agusto & Tchuenche (2013)
*μ*_*m*_	0.078	[0, 0.1]	Adeniyi et al. (2023), Agusto & Tchuenche (2013)
Λ_*m*_	29000	[15600, 31200]	Chamchod et al. (2011)
*ν*_*m*_	0.09	[0, 0.1]	Akowe et al. (2025), Agusto & Tchuenche (2013)
*δ*_*h*_	0.0009	[0, 0.01]	Adeniyi et al. (2023)
*ρ*	MCMC	[0, 1]	Aluh et al. (2025)
*p*	MCMC	[0, 1]	Haringo et al. (2025)
*κ*	MCMC	[0.01, 0.2]	Akowe et al. (2025)
*σ*_*m*_	MCMC	[0.005,0.02]	Akowe et al. (2025)
*σ*_*t*_	MCMC	[0.00001, 0.003]	Akowe et al. (2025)
*ξ*	MCMC	[0.0017, 0.01]	Agusto & Tchuenche (2013)

To identify these parameters, we employ the Markov Chain Monte Carlo (MCMC) method with a Metropolis-within-Gibbs sampler. Let ***θ*** = (*σ*_*m*_, *σ*_*t*_, *κ*, *ξ*, *p*, *ρ*) be the vector of target parameters to be estimated. The posterior distribution of these parameters given the data **D** is denoted by *P*(***θ***|**D**), which is derived by the Bayes’ theorem

P(θ|D)∝L(D|θ)⋅π(θ),

where *π*(***θ***) represents the prior distributions based on pre-existing epidemiological knowledge before observing the data **D**, *L*(**D**|***θ***) is the likelihood function describing the probability of observing the epidemiological data given the model parameters. In the absence of strong empirical data to inform precise parameter values, we assign independent uniform prior distributions to each model parameter, the ranges of which are shown in Table 2. Since the data are aggregated annual counts of malaria cases, a negative binomial distribution is used to account for the potential overdispersion inherent in the incidence data. The Metropolis-within-Gibbs sampling algorithm is employed to efficiently explore the posterior distribution. The MCMC chain is run for 50, 000 iterations with the first 30, 000 discarded as burn-in. The point and 95% credible interval estimation for fitted parameters are listed in Table 3.

**Table 3:** Parameter values for point estimation and 95% interval estimation.

Parameter	Point estimation	95% confidence interval
*ρ*	0.03	[0.0103, 0.0300]
*p*	0.1	[0.0104, 0.1196]
*κ*	0.12	[0.0960, 0.1942]
*σ*_*m*_	0.012	[0.0108, 0.0196]
*σ*_*t*_	0.000019	[0.0000016, 0.00001916]
*ξ*	0.0018	[0.0017, 0.0020]

## 3 Results

### 3.1 Model calibration

Based on the estimated parameters from MCMC method, model calibration is performed against the clinical malaria incidence reported in Nigeria between January 2015 and December 2024. In model (1), the instantaneous rate at which new cases enter the symptomatic infectious compartment is

$$j_{\mathrm{case}}\left(t;\theta \right)=\left(1-p\right)\left[\nu_{hm}E_{hm}\left(t;\theta \right)+\nu_{ht}E_{ht}\left(t;\theta \right)\right],$$

where ***θ*** = (*σ*_*m*_, *σ*_*t*_, *κ*, *ξ*, *p*, *ρ*) denotes the vector of parameters estimated in the MCMC method. Therefore, the model-predicted number of symptomatic cases during calendar year *k* is calculated as

$$C_{k}\left(\theta \right)=\int_{t_{k-1}}^{t_{k}}j_{\mathrm{case}}\left(t;\theta \right)dt,$$

where *t*_*k*_ − *t*_*k*−1_ = 365 days. The cumulative cases (shown in Fig. 2(b)) is obtained by cumulatively summing the annual observed and model-predicted cases, which are not included in the likelihood and presented as an additional graphical comparison.

**Fig. 2:**
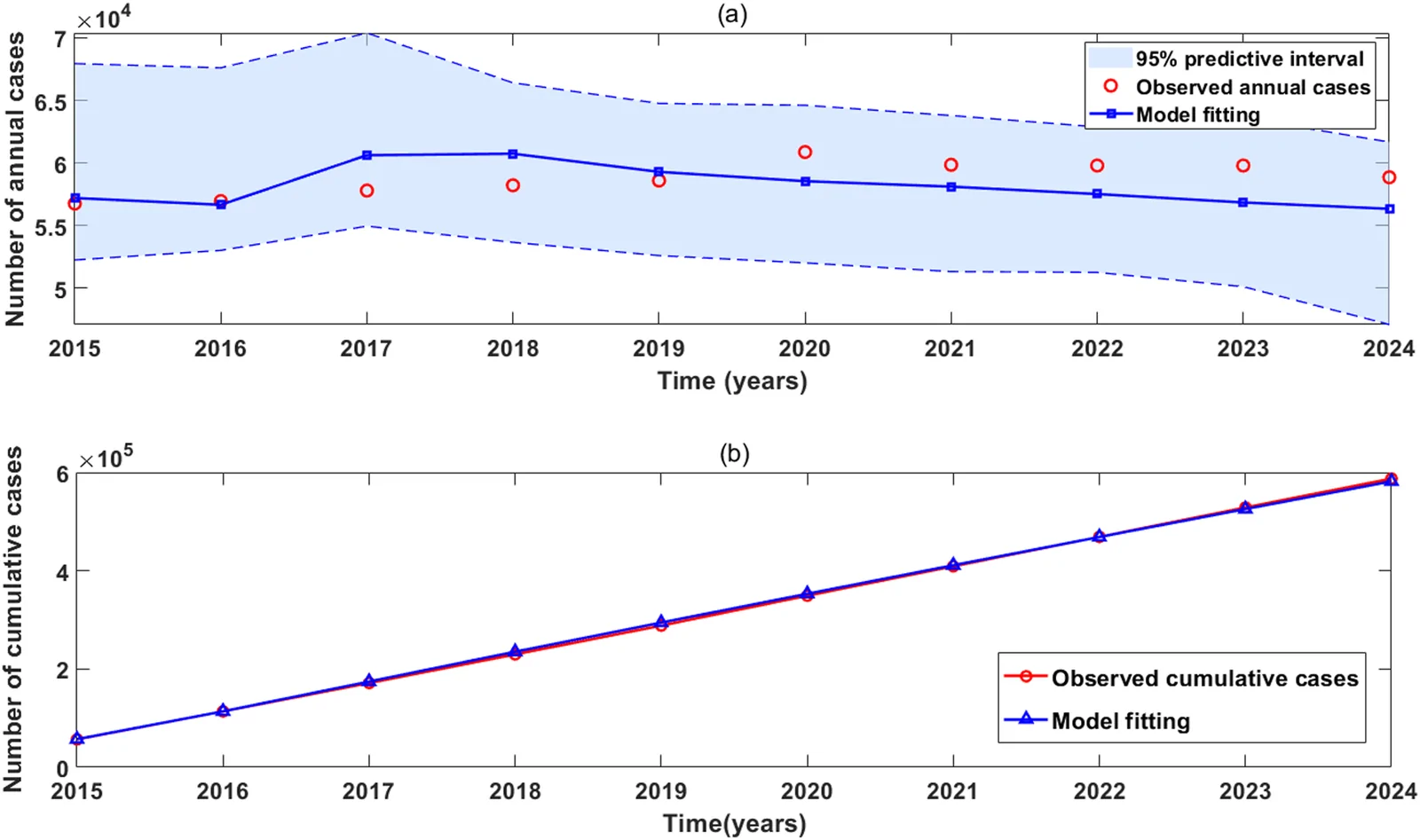
The number of annual and cumulative malaria cases during 2015-2024 in Nigeria and model fitted curves.

The comparison between observed annual/cumulative malaria cases and model-fitted estimates with their 95% predictive intervals for Nigeria from 2015 to 2024 is presented in Fig. 2.

The temporal evolution of annual malaria incidence is displayed in Fig. 2(a). The observed surveillance data (red circles) reveals a relatively stable transmission trend. The model fitting (blue line) successfully tracks the overall trend. Critically, every observed annual case falls within the 95% predictive interval (shaded blue region bounded by dashed lines). Fig. 2(b) presents the corresponding cumulative case counts (red circles) alongside the fitted cumulative projections (blue triangles) based on model (2.1). Over the decade, observed cumulative cases exhibit a highly linear and consistent upward trajectory. The blue triangular markers on the fitted curves are nearly perfectly overlapping the red data points throughout the entire 2015-2024 window. This implies that the vector-host model incorporating transfusion transmission is accurate at capturing the fundamental transmission rate and the overall burden of the disease over long term.

### 3.2 Global sensitivity analysis of *R*_0_

A sensitivity analysis of the basic reproduction number *R*_0_ is performed to identify the key epidemiological parameters driving malaria transmission and to evaluate potential control strategies. This analysis evaluates how changes in model parameters influence the magnitude of *R*_0_, which are visualized in Fig. 3. The values of partial rank correlation coefficient (PRCC) for model parameters offer a quantitative ranking of their relative impact on *R*_0_. Parameters with strongest influence on *R*_0_ are mosquito mortality rate (*μ*_*m*_) and human recovery rate from infection (*γ*_*h*_), which emphasizes the effectiveness of vector control measures and clinical interventions improving patient recovery in disrupting malaria transmission. Parameters associated with blood transfusion including the fraction of asymptomatic individuals (*p*), the proportion of asymptomatic humans who involve blood transfusion (*ρ*) and the transfusion transmission rate (*σ*_*t*_), show relative strong positive influence on *R*_0_, which highlights the critical role of transfusion safety in malaria control. Notably, the sensitivity index of transfusion-transmitted rate *σ*_*t*_ is higher than that of mosquito transmission rate *σ*_*m*_. This result indicates that, from a global statistical perspective, the basic reproduction number *R*_0_ is more sensitive to perturbations in the transfusion-transmitted route than to perturbations in mosquito-mediated transmission pathway. This is biologically reasonable as the transfusion route provides a direct portal for infectious individuals to bypass the mosquito life-cycle and immediately initiate new human infections.

**Fig. 3:**
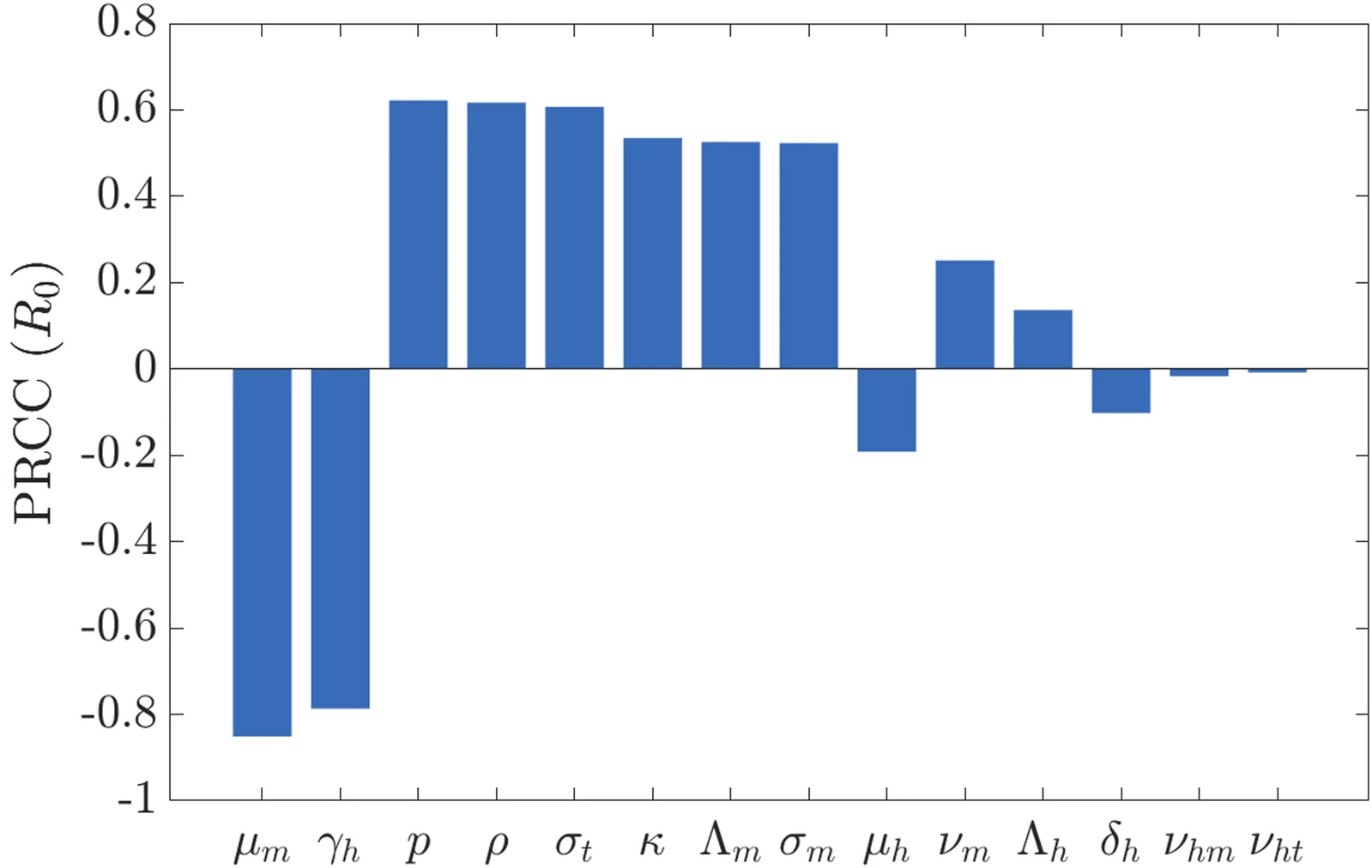
Sensitivity analysis of the basic reproduction number, *R*_0_.

### 3.3 Dynamics of malaria infections to different *σ*_*m*_ and *σ*_*t*_

We next investigate the separable impact of the two distinct transmission pathways (i.e., mosquito-borne and transfusion-transmitted) on the dynamics of malaria infections, which consist of exposed humans via mosquito bites (*E*_*hm*_), exposed humans via blood transfusion (*E*_*ht*_), infectious humans (*I*_*h*_) and asymptomatic humans (*A*_*h*_). Fig. 4, Fig. 5 illustrate the temporal evolution of the infected compartments under four different values of *σ*_*m*_ and *σ*_*t*_ separately. Notably, either mosquito transmission rate (*σ*_*m*_) or transfusion-transmitted rate (*σ*_*t*_) aggravates the overall disease burden in the population, that is, all infected subgroups exhibit more intense epidemic peaks with higher amplitudes that occur earlier in time. Specifically, the exposed humans infected by mosquito bites (*E*_*hm*_) shows the most substantial surge when the mosquito transmission rate (*σ*_*m*_) increases (see the top-left panel of Fig. 4). Besides, increased *σ*_*m*_ significantly elevates the peak of exposed humans infected via blood transfusion *E*_*ht*_ (see the top-right panel of Fig. 4). Similarly, increasing the transfusion-transmitted rate *σ*_*t*_ has a direct effect on *E*_*ht*_, presented in the top-right panel of Fig. 5, where higher *σ*_*t*_ values lead to a significantly larger and more prolonged peak of transfusion-transmitted infections. While the impact of *σ*_*t*_ on the mosquito-driven components (*E*_*hm*_ and *I*_*h*_) is relatively milder compared to that of *σ*_*m*_.

**Fig. 4:**
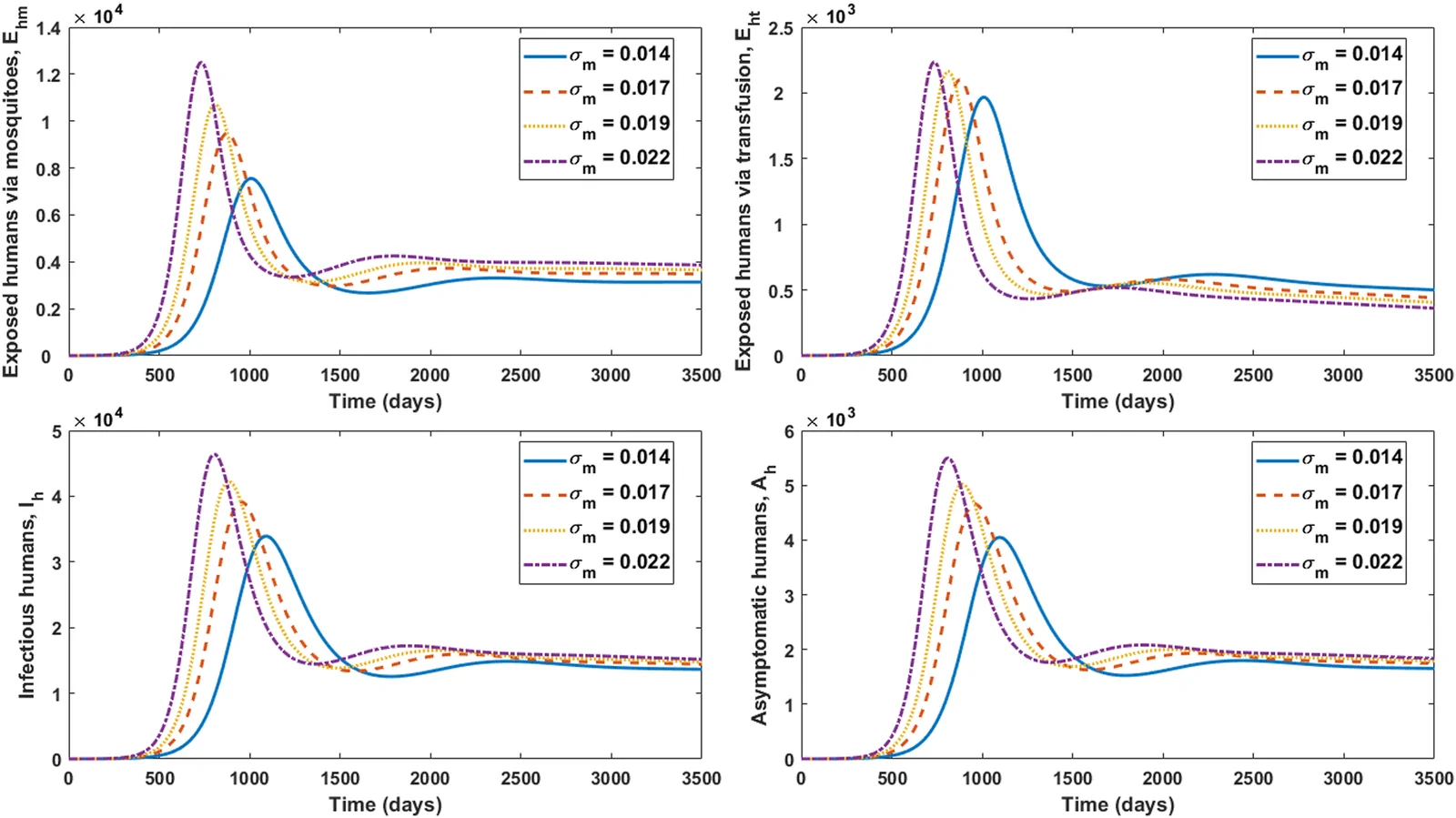
Dynamics of infected compartments (*E*_*hm*_, *E*_*ht*_, *I*_*h*_, *A*_*h*_) under different mosquito-borne transmission rate, *σ*_*m*_. Other parameters are fixed as specified in Table 2.

**Fig. 5:**
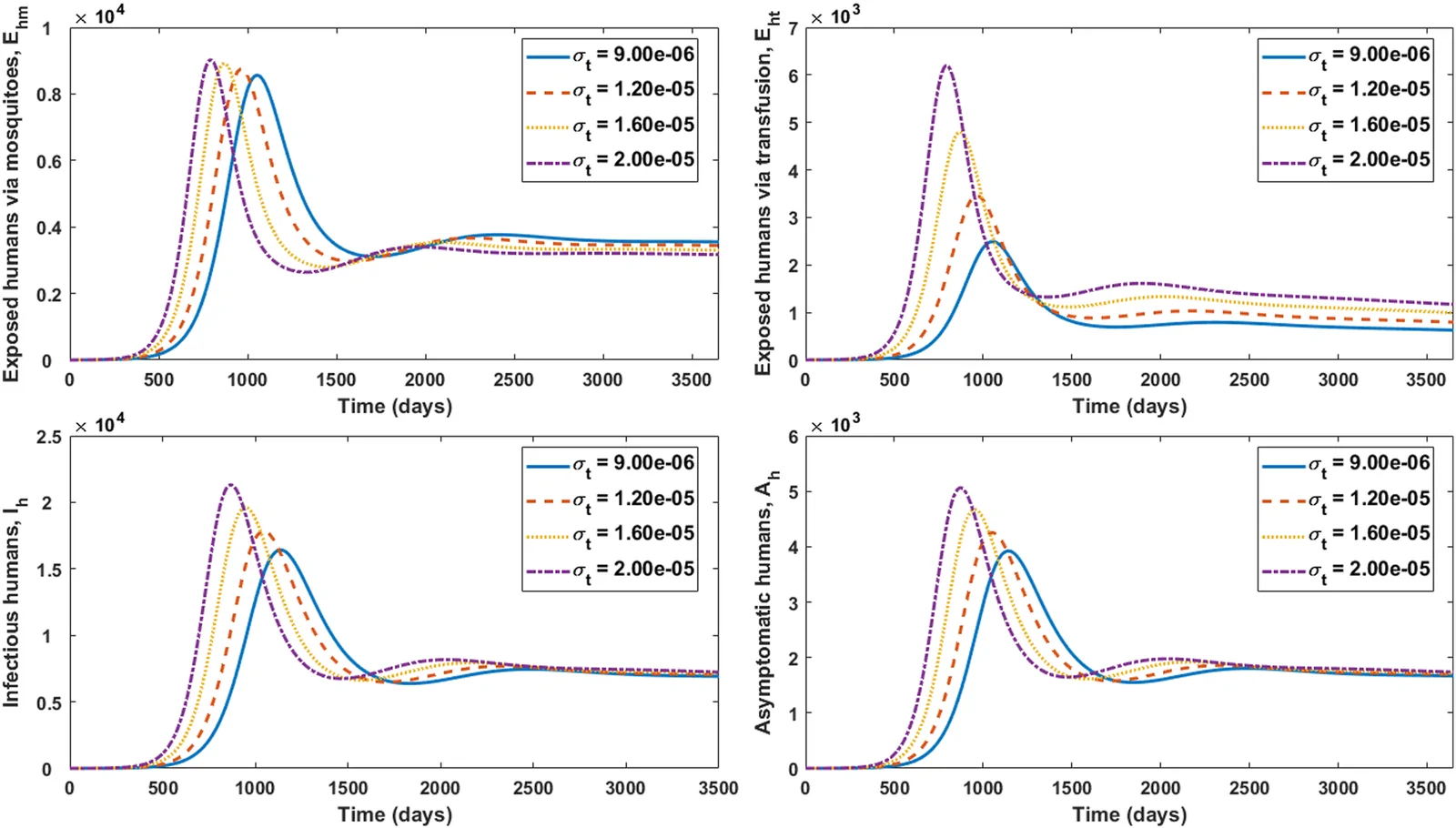
Dynamics of infected compartments (*E*_*hm*_, *E*_*ht*_, *I*_*h*_, *A*_*h*_) under different blood transfusion transmitted rate, *σ*_*t*_. Other parameters are fixed as specified in Table 2.

### 3.4 Combined effects of *σ*_*m*_ and *σ*_*t*_

Fig. 4, Fig. 5 provide valuable insights into the transient dynamics of exposed and infectious populations under the variations of single parameter. In natural transmission settings, both mosquito-mediated transmission (*σ*_*m*_) and transfusion-transmitted infection (*σ*_*t*_) act concurrently. Therefore, relying solely on single-parameter perturbation curves fails to fully elucidate the relative contribution of each route to the overall outbreak potential. To bridge this gap, a bi-variate parameter sensitivity analysis is conducted utilizing contour plots. Fig. 6 illustrates the simultaneous impact of varying both *σ*_*m*_ and *σ*_*t*_ on three key epidemiological indices, which are the basic reproduction number (*R*_0_), the peak size of infectious humans (*I*_*h*_) and the time to reach this peak. As expected, the contour plots demonstrate that both *R*_0_ and the peak size of malaria infections increase while the peak time decreases as either parameter increases. The contour lines display a strong diagonal trend, suggesting an additive relationship where simultaneous increases in vector-borne and transfusion-mediated transmission drastically elevate the epidemic potential.

**Fig. 6:**
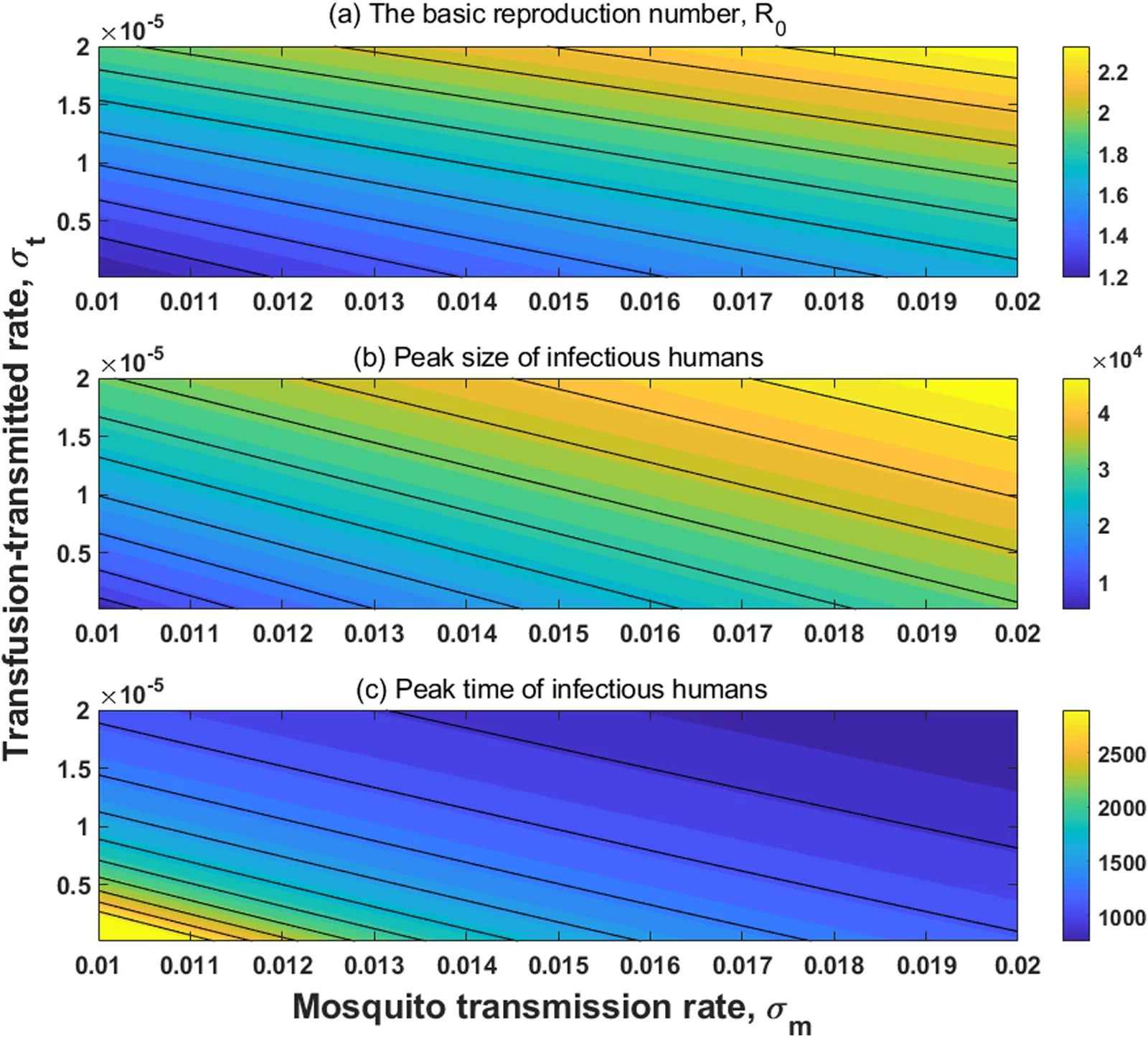
Integrated effects of mosquito transmission rate (*σ*_*m*_) and transfusion transmitted rate (*σ*_*t*_) on (a) the basic reproduction number, *R*_0_, (b) the peak size of symptomatic infectious humans, (c) the peak time of infectious humans.

### 3.5 Relative contribution of transfusion-mediated transmission to *R*_0_

The contour plot in Fig. 7 illustrates the relative contribution of transfusion-mediated transmission to the basic reproduction number, *H*_*t*_ (defined in (2.5)), as the mosquito-borne transmission rate *σ*_*m*_ and the transfusion-mediated transmission rate *σ*_*t*_ vary. Overall, *H*_*t*_ increases monotonically with *σ*_*t*_ and decreases as *σ*_*m*_ increases. The thick black line represents *H*_*t*_ = *H*_*m*_ = 50%, marking the parameter combinations where both transmission pathways contributes equally. Above this threshold, transfusion-mediated transmission contributes more than 50% to *R*_0_, thereby emerging as the dominant mode of transmission. In contrast, mosquito-borne transmission predominates blow the same threshold. It is also worth noting that *H*_*t*_ varies approximately from 20% to 65%, reaching its highest value when *σ*_*t*_ is large and *σ*_*m*_ is small, which indicates that transfusion-mediated transmission can substantially shape the transmission potential even after mosquito control measures have reduced *σ*_*m*_ to low levels. These results emphasize the importance of interventions that reduce *σ*_*t*_, such as blood screening and transfusion safety measures.

**Fig. 7:**
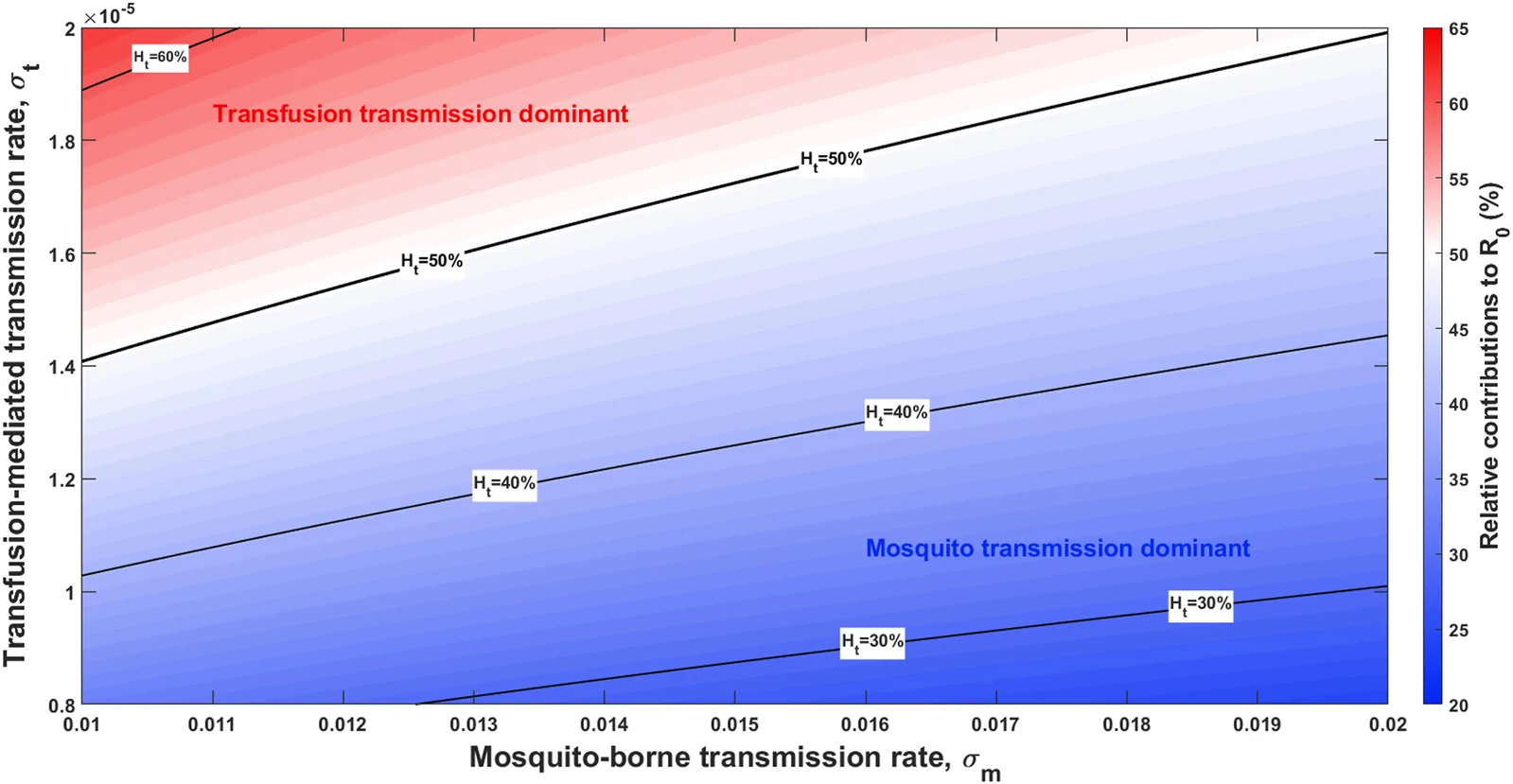
Relative contributions of the transfusion-mediated transmission, *H*_*t*_ to *R*_0_.

### 3.6 Relative contribution of transfusion-mediated transmission to new infection flows

Fig. 8(a) shows the route-specific new human infection flows generated by mosquito-borne transmission (*J*_*m*_(*t*), defined in (2.6)) and transfusion-mediated transmission (*J*_*t*_(*t*), defined in (2.7)), together with the total human infection flow (*J*_*m*_(*t*) + *J*_*t*_(*t*)). Both routes display a pronounced epidemic peak, while the mosquito-borne infection flow reaches a higher peak than the transfusion-mediated flow. After the main peak, both infection flows decline rapidly to a lower persistent level. In addition, the mosquito-borne component remains consistently larger than the transfusion-mediated component throughout most of the simulation period. Fig. 8(c) presents the cumulative route-specific new infection flows, the growth pattern of which is closely related to the instantaneous dynamics in Fig. 8(a). As expected, all the cumulative curves increase monotonically while the cumulative mosquito-borne infection burden remains consistently higher than the cumulative transfusion-mediated burden.

**Fig. 8:**
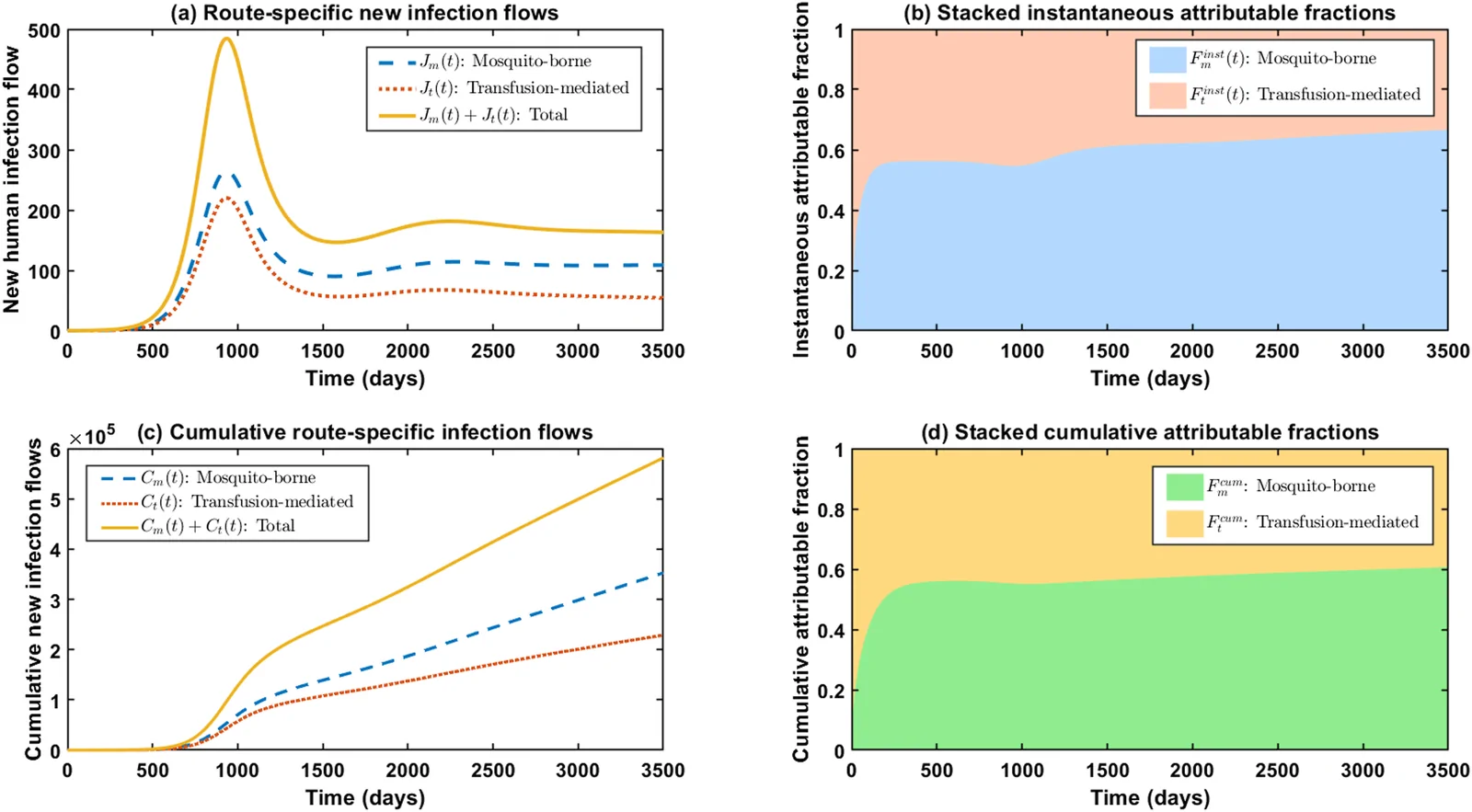
Relative contributions of the transfusion-mediated transmission to the new infection flow.

Fig. 8(b) and (d) represent respectively the corresponding instantaneous and cumulative attributable fractions of each transmission route. The stacked area plot shows that both the instantaneous and cumulative mosquito-borne attributable fraction rapidly increases during the early phase and then remains above 0.5 for almost the entire period. The instantaneous attributable fraction, $F_{m}^{\text{inst}}\left(t\right)$ (defined in (2.9)) increases to approximately 0.66 at the end of the simulation while $F_{t}^{\text{inst}}$ (defined in (2.8)) decline to about 0.34. In contrast, the cumulative mosquito-borne fraction at the final time is lower, around 0.6, and the cumulative transfusion-mediated fraction is higher, around 0.4. Although mosquito-borne transmission is dominant in both analyses, the cumulative perspective emphasizes that transfusion-mediated transmission has made a substantial long-term contribution to the total number of infections.

Fig. 9(a) compares the directly accumulated transfusion-mediated infection burden, *C*_*t*_(*T*) (defined in (2.10)), with the counterfactual burden averted by eliminating transfusion-mediated transmission, Δ*C*_*t*_(*T*), defined in (2.12). Throughout the simulation period, Δ*C*_*t*_(*T*) remains substantially larger than *C*_*t*_(*T*), the gap between which becomes especially pronounced after the main epidemic growth phase. This indicates that the total number of infections preventable by removing transfusion-mediated transmission exceeds the number of infections directly generated through the transmission route. Fig. 9(b) further supports this conclusion by comparing the direct cumulative attributable fraction, $F_{t}^{\text{cum}}\left(t\right)$ (defined in (2.11)), with the counterfactual attributable fraction, $AF_{t}^{\text{cf}}\left(t\right)$ (defined in (2.13)). Over the entire time interval, $AF_{t}^{\text{cf}}\left(t\right)$ remains markedly higher than $F_{t}^{\text{cum}}\left(t\right)$. In addition, $AF_{t}^{\text{cf}}\left(t\right)$ initially rises to nearly 1 during the early epidemic peak and then gradually declines to approximately 0.57 at the final time, which is still higher than the final direct cumulative attributable fraction.

**Fig. 9:**
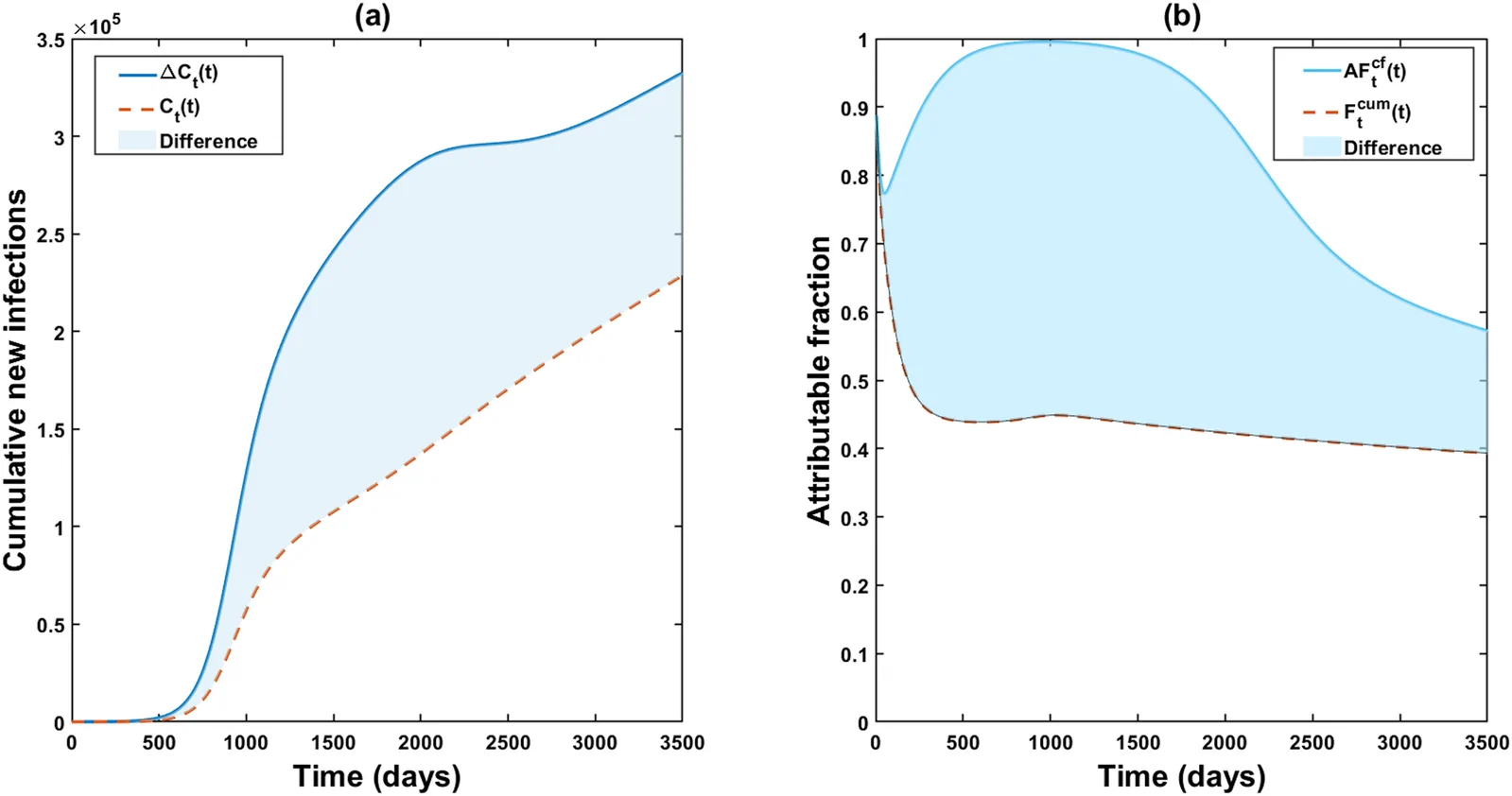
(a) Direct and counterfactual transfusion-attributable infection burdens. (b) Direct cumulative fraction and counterfactual transfusion-attributable fraction.

These results show that the direct route-specific decomposition underestimates the epidemiological importance of transfusion-mediated transmission. Previous analysis of new infection flows indicate that mosquito-borne transmission is the dominant direct contributor, whereas transfusion-mediated transmission accounts a smaller but persistent share. However, the counterfactual comparison demonstrates that the impact of transfusion-mediated transmission is amplified since infections generated through transfusion can initiate chains of later secondary cases infected by mosquito-borne transmission. Consequently, the counterfactual attributable fraction $AF_{t}^{\text{cf}}\left(t\right)$ provides a broader measure of public-health impact than the direct cumulative fraction $F_{t}^{\text{cum}}\left(t\right)$. These findings suggest that interventions targeting transfusion-mediated transmission may yield benefits greater than those inferred from direct route-specific infection counts alone.

While the preceding simulations quantify the magnitude of transfusion-mediated contribution under the baseline parameter values, they do not indicate which parameters are primarily responsible for the observed differences between the direct cumulative burden *C*_*t*_(*T*), the direct cumulative attributable fraction $F_{t}^{\text{cum}}\left(t\right)$ and the counterfactual attributable fraction $AF_{t}^{\text{cf}}\left(t\right)$. To complement the above analysis, a partial rank correlation coefficient analysis (PRCC) is performed to assess the sensitivity of these quantities to the transmission-related parameters *κ*, *σ*_*m*_, *σ*_*t*_, *p*, *ρ*. The PRCC results in Fig. 10 show that the transfusion-related transmission parameter *σ*_*t*_ is dominant positive driver of all three transfusion-related burden measures. This indicates that increasing the efficiency of transfusion-mediated transmission directly increases both the absolute cumulative number of transfusion-generated infections and the relative importance of this route. Beside, parameters *p* and *ρ* display strong positive correlations with *C*_*t*_(*T*) and $F_{t}^{\text{cum}}$ but weaker positive correlation with $AF_{t}^{\text{cf}}\left(t\right)$, which indicates that parameters enhancing transfusion-related exposure can substantially increase the observed contribution of the transmission route. In contrast, *σ*_*m*_ and *κ* have negative association with these three quantities while the effects on *C*_*t*_(*T*) is relative weaker. This means that stronger mosquito-borne transmission reduces the relative importance of transfusion-mediated transmission, which is consistent with previous indication that mosquito-borne transmission is the dominant contribution to new infections.

**Fig. 10:**
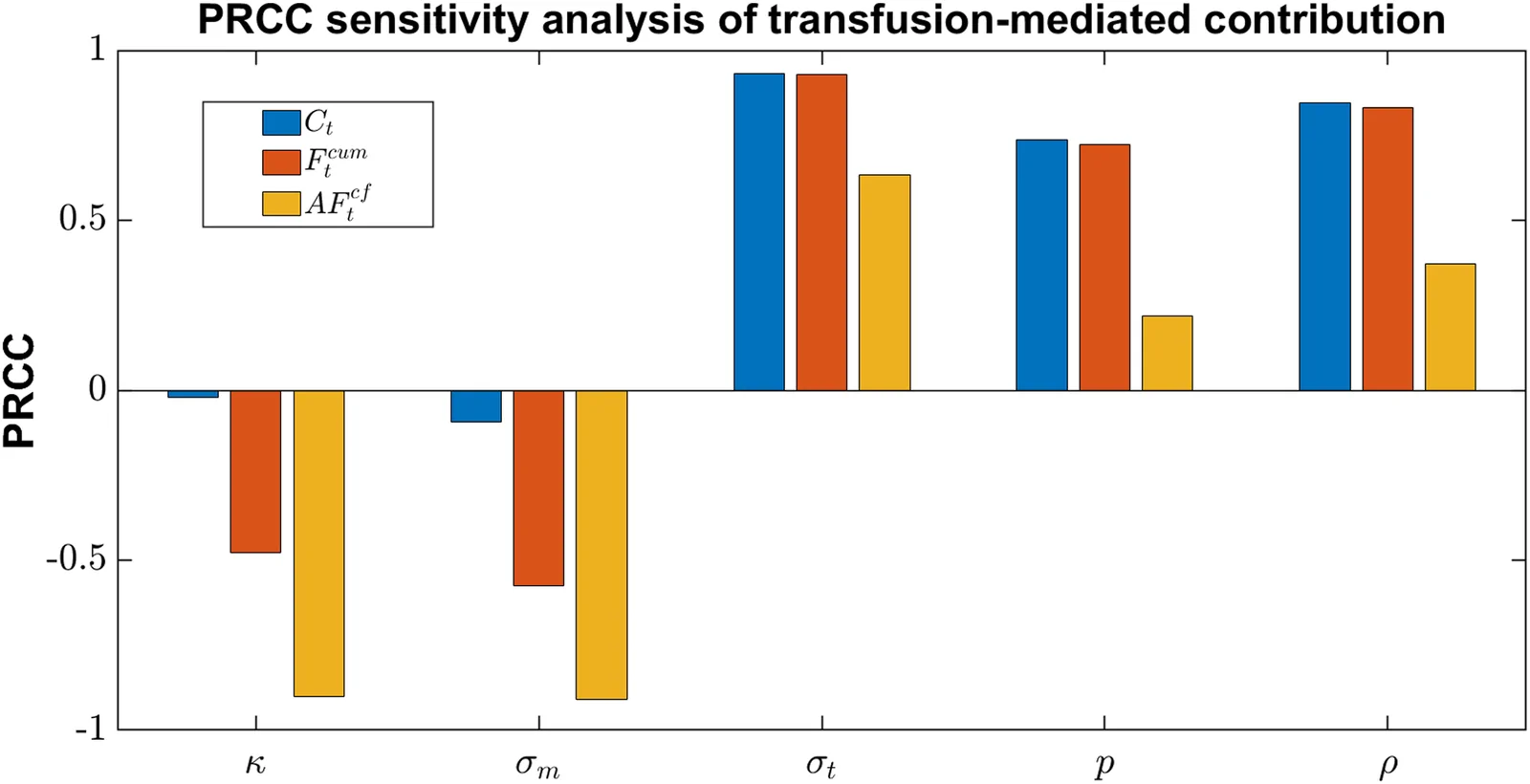
Partial rank correlation coefficients (PRCCs) illustrating the sensitivity of transfusion-mediated contribution metrics to key transmission parameters.

As an illustrative translation of the model findings, we further estimate the annual number of preventable infections if routine malaria screening for blood donors is implemented. Based on the minimum clinical demand of 1.8 × 10^6^ blood units per year estimated in (Iro et al., 2019), and assuming that this demand is fully met and all units are transfused, the number of blood units that are actually transfused is therefore approximately 1.8 × 10^6^. Applying the donor paraitemia prevalence of 21.1% provided in (Olopade et al., 2024), roughly 379, 800 potentially parasitemic units would be expected among 1.8 million donated blood units in the absence of screening. Assuming 95% screening sensitivity, full screening coverage and effective blood unit-to-recipient transmission probability of 50%, approximately 180, 405 direct transfusion-transmitted infections could be detected and excluded annually. Furthermore, the overall avertable burden would be even larger since preventing these infections would also interrupt subsequent mosquito-borne transmission, as indicated by the counterfactual analysis in Fig. 9(b).

In summary, the mosquito-borne route acts as the primary driver of initial epidemic waves while the blood-transfusion route as a persistent secondary transmission pathways maintains the infection in asymptomatic individuals and potentially causing infections even after mosquito control measures have reduced *σ*_*m*_ to low levels. These findings highlight the critical importance of comprehensive screening protocols for blood transfusion, especially in malaria endemic regions where a substantial proportion of the population may harbor asymptomatic parasitemia (*A*_*h*_).

## 4 Discussion

Classical vector-host malaria models primarily attribute new human infections to mosquito-borne transmission. However, endemic regions with substantial numbers of asymptomatic carriers and unsafe transfusion practices can introduce infection directly into the human population bypassing the mosquito cycle. In this paper, a novel mathematical model for malaria transmission is proposed and analyzed by explicitly incorporating both mosquito-borne and transfusion-mediated routes. Based on this model, we provide rigorous definitions of relative contributions of transfusion-mediated transmission to the basic reproduction number and new infection flows. By leveraging 10 years of malaria incidence data of Nigeria and employing Markov Chain Monte Carlo (MCMC) method, key transmission parameters are estimated. The model fitting results show an excellent agreement between the observed annual/cumulative malaria cases and model simulations, which provides a robust foundation for subsequent sensitivity and dynamical analysis.

The Partial Rank Correlation Coefficient (PRCC) analysis identifies the most influential parameters on the basic reproduction number *R*_0_. Consistent with previous modeling researches on malaria transmission, the mosquito mortality rate, the human recovery rate exhibit the strongest negative correlations with *R*_0_. Notably, this PRCC analysis reveals that the transfusion-transmitted rate (*σ*_*t*_) exhibits a higher positive PRCC value compared to mosquito transmission rate (*σ*_*m*_). This may be explained by the direct nature of transfusion-mediated infection, which bypasses the mosquito life cycle and immediately introduces infection into the human population. Consequently, small variations in the transfusion transmission rate (*σ*_*t*_) can trigger significant changes in the *R*_0_ value. The time-series simulations reveal that increasing either *σ*_*m*_ or *σ*_*t*_ intensifies epidemic dynamics by producing higher and earlier peaks in exposed, infectious and asymptomatic human populations. Particularly, mosquito-borne transmission appears to have a stronger influence on the overall epidemic peak compared to transfusion-mediated transmission. Bi-variate contour analysis further demonstrates that higher transmission rates in both routes lead to larger *R*_0_, greater peak number of infectious humans and an earlier epidemic peak.

Moreover, the route-specific and counterfactual analyses reveals a key finding. Even through transfusion-mediated transmission accounts for a smaller direct attributable fraction of new infections compared to mosquito-borne transmission, the overall epidemiological impact of transfusion-mediated transmission can be substantially larger. In particular, the counterfactual attributable fraction indicates that eliminating transfusion-mediated transmission could avert a larger fraction of the total infection burden than suggested by direct route-specific contribution analysis.

These results have significant implications for malaria control strategies, particularly in endemic regions with high transfusion rates or unsafe blood supplies. Interventions targeting mosquito populations (e.g., insecticide-treated nets, indoor residual spraying) remain the most effective tools for delaying epidemic peaks, preventing healthcare system saturation, and reducing overall mortality. The non-negligible roles of transfusion-mediated transmission route suggest that malaria control strategies should not rely exclusively on vector-control interventions, but also incorporate blood-safety measures such as donor screening and improved transfusion management. Overall, the numerical simulations highlight the importance of integrating both vector-focused and transfusion-focused interventions in order to reduce malaria transmission and prevent avoidable infection burden.

In conclusion, this comprehensive data-based modeling analysis provides a quantitative framework for understanding the dual transmission pathways of malaria. This study has several limitations. First, parameter estimates rely on the temporal averaging of data from Nigeria, and the applicability of these findings to other geographical regions requires validation. Besides, the model assumes homogeneous mixing and does not incorporate seasonal effects on mosquitoes, which may affect the precise timing of epidemic peaks. Future work could extend the model to seasonal malaria model to explore the impact of varying temperature and precipitation on the transmission dynamics of malaria.

## CRediT authorship contribution statement

**Kaihui Liu:** Writing – review & editing, Writing – original draft, Visualization, Methodology, Formal analysis, Conceptualization. **Wen Shi:** Writing – review & editing, Visualization, Validation, Software, Data curation.

## Declaration of competing interest

The authors declare that they have no known competing financial interests or personal relationships that could have appeared to influence the work reported in this paper.

## Appendix Derivation of the basic reproduction number, *R*_0_

It is easy to deduce the disease-free equilibrium (DFE) by setting the right-hand side of the equations to zero in system (2.1).

$$\left\{\begin{array}{l} \Lambda_{m}-\kappa \frac{I_{h}+A_{h}}{N_{h}}S_{m}-\mu_{m}S_{m}=0\\ \;\kappa \frac{I_{h}+A_{h}}{N_{h}}S_{m}-\nu_{m}E_{m}-\mu_{m}E_{m}=0\\ \;\nu_{m}E_{m}-\mu_{m}I_{m}=0\\ \;\Lambda_{h}-\sigma_{m}\frac{I_{m}}{N_{h}}S_{h}-\sigma_{t}\rho A_{h}S_{h}-\mu_{h}S_{h}+\xi R_{h}=0\\ \;\sigma_{m}\frac{I_{m}}{N_{h}}S_{h}-\nu_{hm}E_{hm}-\mu_{h}E_{hm}=0\\ \;\sigma_{t}\rho A_{h}S_{h}-\nu_{ht}E_{ht}-\mu_{h}E_{ht}=0\\ \;p\nu_{hm}E_{hm}+p\nu_{ht}E_{ht}-\left(\mu_{h}+\gamma_{h}\right)A_{h}=0\\ \;\left(1-p\right)\nu_{hm}E_{hm}+\left(1-p\right)\nu_{ht}E_{ht}-\left(\mu_{h}+\delta_{h}+\gamma_{h}\right)I_{h}=0\\ \;\gamma_{h}\left(I_{h}+A_{h}\right)-\mu_{h}R_{h}-\xi R_{h}=0 \end{array}\right)$$

Thus, the disease-free equilibrium (DFE) is given as follows

$$E_{0}=\left(S_{m}^{*},\;0,\;0,\;S_{h}^{*},\;0,\;0,\;0,\;0\right),$$

where $S_{m}^{*}=\frac{\Lambda_{m}}{\mu_{m}}$, $S_{h}^{*}=\frac{\Lambda_{h}}{\mu_{h}}$ and $N_{h}^{*}=S_{h}^{*}$. Then, the basic reproduction number is calculated by employing the next generation matrix approach van den Driessche and Watmough (2002).

Let $x={\left[E_{m},I_{m},E_{hm},E_{ht},A_{h},I_{h}\right]}^{T}$, the system involving infected compartments can be written as

$$\frac{dx}{dt}=F\left(x\right)-V\left(x\right),$$

where

$$\begin{array}{l} F\left(x\right)=\left[\begin{array}{l} \kappa \frac{I_{h}+A_{h}}{N_{h}}S_{m}\\ 0\\ \sigma_{m}\frac{I_{m}}{N_{h}}S_{h}\\ \sigma_{t}\rho A_{h}S_{h}\\ 0\\ 0 \end{array}\right],V\left(x\right)=\left[\begin{array}{l} \nu_{m}E_{m}+\mu_{m}E_{m}\\ -\nu_{m}E_{m}+\mu_{m}I_{m}\\ \mu_{h}E_{hm}+\nu_{hm}E_{hm}\\ \mu_{h}E_{ht}+\nu_{ht}E_{ht}\\ \left(\mu_{h}+\gamma_{h}\right)A_{h}-p\nu_{hm}E_{hm}-p\nu_{ht}E_{ht}\\ \left(\mu_{h}+\delta_{h}+\gamma_{h}\right)I_{h}-\left(1-p\right)\nu_{hm}E_{hm}-\left(1-p\right)\nu_{ht}E_{ht} \end{array}\right]. \end{array}$$

Linearising at the DEF (*E*_0_) yields the Jacobian matrices of *F* and *V* as follows

$$F=\left(\begin{array}{llllll} 0 & 0 & 0 & 0 & \kappa \frac{S_{m}^{*}}{N_{h}^{*}} & \kappa \frac{S_{m}^{*}}{N_{h}^{*}}\\ 0 & 0 & 0 & 0 & 0 & 0\\ 0 & \sigma_{m} & 0 & 0 & 0 & 0\\ 0 & 0 & 0 & 0 & \sigma_{t}\rho \frac{\Lambda_{h}}{\mu_{h}} & 0\\ 0 & 0 & 0 & 0 & 0 & 0\\ 0 & 0 & 0 & 0 & 0 & 0 \end{array}\right)$$

and

$$V=\left(\begin{array}{llllll} \nu_{m}+\mu_{m} & 0 & 0 & 0 & 0 & 0\\ -\nu_{m} & \mu_{m} & 0 & 0 & 0 & 0\\ 0 & 0 & \mu_{h}+\nu_{hm} & 0 & 0 & 0\\ 0 & 0 & 0 & \mu_{h}+\nu_{ht} & 0 & 0\\ 0 & 0 & -p\nu_{hm} & -p\nu_{ht} & \mu_{h}+\gamma_{h} & 0\\ 0 & 0 & -\left(1-p\right)\nu_{hm} & -\left(1-p\right)\nu_{ht} & 0 & \mu_{h}+\delta_{h}+\gamma_{h} \end{array}\right).$$

By easy calculation, the next generation matrix is

$$FV^{-1}=\left(\begin{array}{llllll} 0 & 0 & K_{13} & K_{14} & K_{15} & K_{16}\\ 0 & 0 & 0 & 0 & 0 & 0\\ K_{31} & K_{32} & 0 & 0 & 0 & 0\\ 0 & 0 & K_{43} & K_{44} & K_{45} & 0\\ 0 & 0 & 0 & 0 & 0 & 0\\ 0 & 0 & 0 & 0 & 0 & 0 \end{array}\right)$$

where

$$\begin{array}{ll} & K_{13}=\frac{\kappa S_{m}^{*}\nu_{hm}}{N_{h}^{*}\left(\mu_{h}+\nu_{hm}\right)}\left(\frac{p}{\mu_{h}+\gamma_{h}}+\frac{1-p}{\mu_{h}+\delta_{h}+\gamma_{h}}\right),\\ & K_{14}=\frac{\kappa S_{m}^{*}\nu_{ht}}{N_{h}^{*}\left(\mu_{h}+\nu_{ht}\right)}\left(\frac{p}{\mu_{h}+\gamma_{h}}+\frac{1-p}{\mu_{h}+\delta_{h}+\gamma_{h}}\right),\\ & K_{15}=\frac{\kappa S_{m}^{*}}{N_{h}^{*}\left(\mu_{h}+\gamma_{h}\right)},\;\;K_{16}=\frac{\kappa S_{m}^{*}}{N_{h}^{*}\left(\mu_{h}+\delta_{h}+\gamma_{h}\right)},\\ & K_{31}=\frac{\sigma_{m}\nu_{m}}{\mu_{m}\left(\mu_{m}+\nu_{m}\right)},\;\;K_{32}=\frac{\sigma_{m}}{\mu_{m}},\;\;K_{43}=\frac{\sigma_{t}\rho S_{h}^{*}p\nu_{hm}}{\left(\mu_{h}+\nu_{hm}\right)\left(\mu_{h}+\gamma_{h}\right)},\\ & K_{44}=\frac{\sigma_{t}\rho S_{h}^{*}p\nu_{ht}}{\left(\mu_{h}+\nu_{ht}\right)\left(\mu_{h}+\gamma_{h}\right)},\;\;K_{45}=\frac{\sigma_{t}\rho S_{h}^{*}}{\mu_{h}+\gamma_{h}}. \end{array}$$

Let

$$R_{0m}=\sqrt{\frac{\kappa S_{m}^{*}}{N_{h}^{*}}\cdot \frac{\nu_{m}}{\mu_{m}+\nu_{m}}\cdot \frac{\sigma_{m}}{\mu_{m}}\cdot \left[p\frac{\nu_{hm}}{\mu_{h}+\nu_{hm}}\frac{1}{\mu_{h}+\gamma_{h}}+\left(1-p\right)\frac{\nu_{hm}}{\mu_{h}+\nu_{hm}}\frac{1}{\mu_{h}+\delta_{h}+\gamma_{h}}\right]},$$

and

$$R_{0t}=\sigma_{t}\rho S_{h}^{*}\cdot \frac{1}{\left(\mu_{h}+\gamma_{h}\right)}\cdot p\frac{\nu_{ht}}{\left(\mu_{h}+\nu_{ht}\right)}.$$

Remark 4.1 Note that the formula of the basic reproduction number, *R*_0_ clearly shows the roles of mosquito-borne and transfusion-mediated route on malaria transmission.

• $\kappa S_{m}^{*}/N_{h}^{*}$ denotes the number of mosquitoes infected by one infectious humans.
• *ν*_*m*_/(*μ*_*m*_ + *ν*_*m*_) is the probability that an exposed mosquito survives the latent period to become infectious.
• *σ*_*m*_/*μ*_*m*_ represents the expected number of human infections caused by one infected mosquito during its average lifespan 1/*μ*_*m*_.
• The term in square brackets describes an exposed human infected via mosquito bites (*E*_*hm*_) becoming either *A*_*h*_ or *I*_*h*_ and surviving from respective period of infectiousness.

Then, *R*_0*m*_ can be defined as the reproduction number of mosquito-borne transmission.

• $\sigma_{t}\rho S_{h}^{*}$ represents the average number of new human infections generated per unit time by one asymptomatic individual *A*_*h*_ via blood transfusion.
• 1/(*μ*_*h*_ + *γ*_*h*_) is the mean infectious period of an asymptomatic individual, *pν*_*ht*_/(*μ*_*h*_ + *ν*_*ht*_) denotes the probability that an individual infected through blood transfusion will eventually become an asymptomatic carrier *A*_*h*_.

Then, *R*_0*t*_ can be defined as the reproduction number of transfusion-mediated transmission.

The eigenvalues of *FV*^−1^ are obtained by solving the following characteristic equation:

$$\lambda^{4}\left(\lambda^{2}-R_{0t}\lambda -R_{0m}^{2}\right)=0.$$

Then, the basic reproduction number *R*_0_, defined as the spectral radius of the next generation matrix *FV*^−1^, is shown as follows

$$R_{0}=\rho \left(FV^{-1}\right)=\frac{1}{2}\left(R_{0t}+\sqrt{R_{0t}^{2}+4R_{0m}^{2}}\right).$$
